# The RNA m6A modification might participate in microglial activation during hypoxic–ischemic brain damage in neonatal mice

**DOI:** 10.1186/s40246-023-00527-y

**Published:** 2023-08-25

**Authors:** Xiaojuan Su, Lingyi Huang, Shiping Li, Junjie Ying, Fengyan Zhao, Shaopu Wang, Qian Liu, Yi Qu, Dezhi Mu

**Affiliations:** 1 Department of Pediatrics/Key Laboratory of Birth Defects and Related Diseases of Women and Children (Ministry of Education), West China Second University Hospital, Sichuan University, Chengdu, 610041 China; 2 West China College of Stomatology/State Key Laboratory of Oral Diseases, Sichuan University, Chengdu, 610041 China

**Keywords:** Hypoxia–ischemia brain damage, Microglia activation, RNA-sequencing, m6A modification, RNA m6A regulators

## Abstract

**Background:**

The RNA m6A modification has been implicated in multiple neurological diseases as well as macrophage activation. However, whether it regulates microglial activation during hypoxic-ischemic brain damage (HIBD) in neonates remains unknown. Here, we aim to examine whether the m6A modification is involved in modulating microglial activation during HIBD. We employed an oxygen and glucose deprivation microglial model for in vitro studies and a neonatal mouse model of HIBD. The brain tissue was subjected to RNA-seq to screen for significant changes in the mRNA m6A regulator. Thereafter, we performed validation and bioinformatics analysis of the major m6A regulators.

**Results:**

RNA-seq analysis revealed that, among 141 m6A regulators, 31 exhibited significant differential expression (FC (abs) ≥ 2) in HIBD mice. We then subjected the major m6A regulators Mettl3, Mettl14, Fto, Alkbh5, Ythdf1, and Ythdf2 to further validation, and the results showed that all were significantly downregulated in vitro and in vivo. GO analysis reveals that regulators are mainly involved in the regulation of cellular and metabolic processes. The KEGG results indicate the involvement of the signal transduction pathway.

**Conclusions:**

Our findings demonstrate that m6A modification of mRNA plays a crucial role in the regulation of microglial activation in HIBD, with m6A-associated regulators acting as key modulators of microglial activation.

**Supplementary Information:**

The online version contains supplementary material available at 10.1186/s40246-023-00527-y.

## Background

Hypoxic–ischemic brain damage (HIBD) is the main cause of neonatal death and neurological disability, mainly due to asphyxia caused by hypoxia–ischemia (HI) during the perinatal period [1]. The incidence of HIBD is around 0.15% in developed countries and up to 2.6% in developing countries [2]. Depending on the severity of HI and the state of brain development, approximately 30% of newborns who survive HIBD will experience permanent neurological sequelae, such as epileptic cerebral palsy, which seriously affects the quality of life, imposing a great burden on the family and society [2]. The pathogenesis of HIBD is complex and has not been fully elucidated, with a lack of effective clinical treatment. Further investigation into the pathogenesis of this disease is necessary to identify therapeutic targets and devise novel treatment strategies.

Currently, HIBD pathogenesis is considered to involve multiple cell types, including neurons, astrocytes, and microglia, with phenomena such as energy depletion, excitatory amino acid toxicity, calcium overload, mitochondrial damage, and a delayed inflammatory response all implicated [3]. Microglial activation is greatly involved in HIBD occurrence as well as progression and is defined by the response to environmental stimuli such as HI [4]. When activated, microglia usually polarize to an M1 or M2 phenotype, which is functionally distinct [5]. M1, also known as classically activated macrophages, primarily expresses surface antigens such as CD16 and CD86 [6]. M1 microglia release pro-inflammatory factors, such as tumor necrosis factor-α(TNF-α), interleukin-1β (IL-1β), IL-6, interferon-γ (IFN-γ), and others, promoting the generation of reactive oxygen species and nitric oxide, which are toxic to neurons and other glial cells [7]. In contrast, M2 alternatively-activated macrophages express surface antigens such as arginase-1 (Arg-1) and CD206, engulf cell fragments or dead neurons, and release anti-inflammatory factors (IL-4, IL-10, and TNF-β) to suppress inflammation and promote neuronal survival [8, 9] (Additional file 1: Fig. S1). Taken together, microglial activation plays a central role in HIBD pathogenesis. However, how microglia are activated in the brains of newborns following HI has not been fully elucidated.

In recent years, the m6-methyladenosine (m6A) modification of mRNA, a key epigenetic regulatory mechanism, has been shown to influence cellular function by regulating gene expression [10]. It is the most abundant post-transcriptional modification of mRNA and is dynamically regulated by a series of enzymes that can be divided into three categories: writers, erasers, and readers [11, 12]. Writers mainly consist of methyltransferase complexes (Mettl3/Mettl14, etc.) that add methyl groups (–CH_3_) to the sixth nitrogen atom of adenine in the pre-mRNA molecule, whereas erasers mainly consist of demethylase enzymes (Fto/Alkbh5, etc.) that strip the –CH_3_ from pre-mRNA [13, 14]. The pre-mRNA is subsequently spliced to form mature mRNA [15]. Finally, the mature mRNA is exported from the nucleus and recognized by readers (Ythdf1/Ythdf2, etc.) that further regulate its translation and degradation [16] (Additional file 2: Fig. S2).

An increasing number of studies have shown that m6A RNA modifications are closely related to the occurrence and development of neurological diseases [13, 17]. For example, a significant decrease in neuronal Fto expression was associated with the occurrence and progression of cerebral ischemia–reperfusion injury in rat models [18]. In addition, the reduced activity of Alkbh5 in astrocytes significantly upregulated m6A RNA levels, leading to astrocyte dysfunction and depression in mice [19]. Both in vitro and in vivo studies have indicated that conditional deletion of Mettl14 in oligodendrocytes results in the abnormal splicing of various transcripts, resulting in aberrant oligodendrocyte maturation and myelination [20]. Recent research on macrophages, which are functionally similar to microglia, showed that deletion of Mettl3 led to the inhibition of macrophage activation [21]. Collectively, these studies suggest that the m6A RNA modification in HIBD may also be involved in microglial activation.

**Fig. 1:**
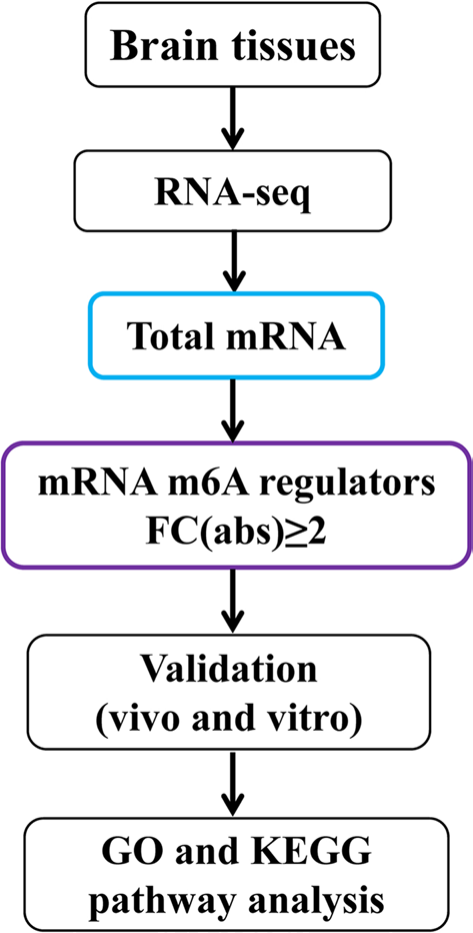
Illustration of the design and procession of the study

To explore this notion, we employed an oxygen and glucose deprivation (OGD) model of primary microglia as well as a HIBD model in newborn C57BL/6 mice. First, we screened the major regulators of the m6A modifications that are significantly altered in HIBD, and validated these in the above models. Regulators were then subjected to Gene Ontology (GO) and Kyoto Encyclopedia of Genes and Genomes (KEGG) enrichment analyses (Fig. 1).

## Results

### The mRNA m6A regulator was significantly altered in activated microglia of neonatal HIBD mice

**Fig. 2:**
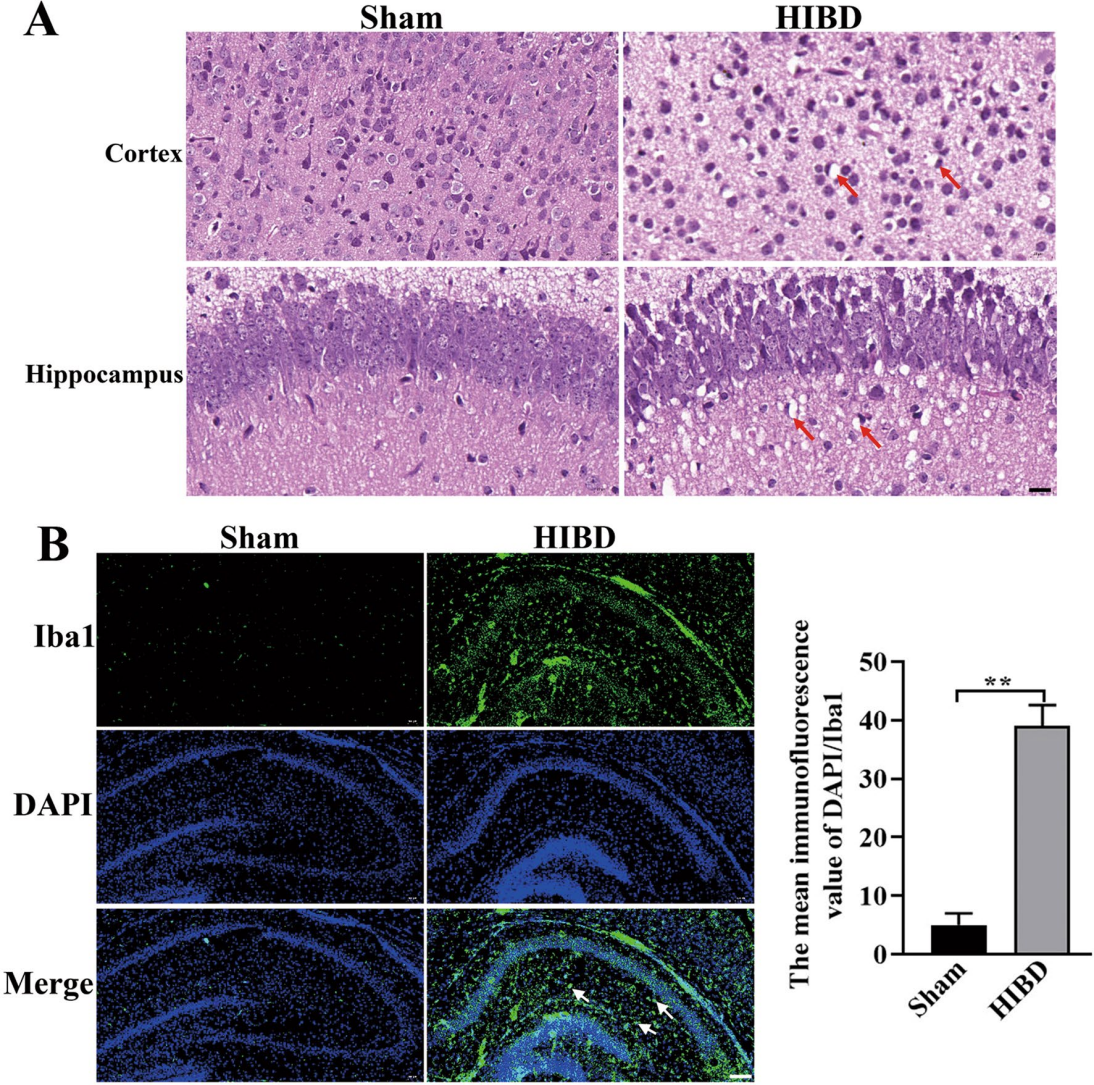
Validation of microglia activation in the HIBD model. **A** H&E was used to evaluate pathological features. Arrows indicate inflammatory cell infiltration. Scale bar: 50 µm. **B** Immunofluorescence staining was used to detect microglia activation in the IBD group and Sham group. DAPI (blue) nuclear staining. Iba1 (green) staining of activated microglia. Scale bar: 100 µm. **P < 0.01

We used the hypoxia–ischemia method to establish HIBD in P9 mice. At P10, we performed H&E staining on mouse brain tissue to validate the HIBD model by evaluating the pathological features. We found mice in the HIBD group shown much more nerve fibers that have been loosely arranged, as well as greater inflammatory cell infiltration, cell edema, and death (Fig. 2A), indicating that the HIBD model was successfully established. Next, we performed Iba1 immunofluorescence staining to examine microglia activation following HIBD. The mean immunofluorescence value of DAPI/Iba1 was significantly higher in the HIBD group than in the Sham group (Fig. 2B). Collectively, these results indicate that we have successfully mimicked the microglial activation of the HIBD model.

**Fig. 3:**
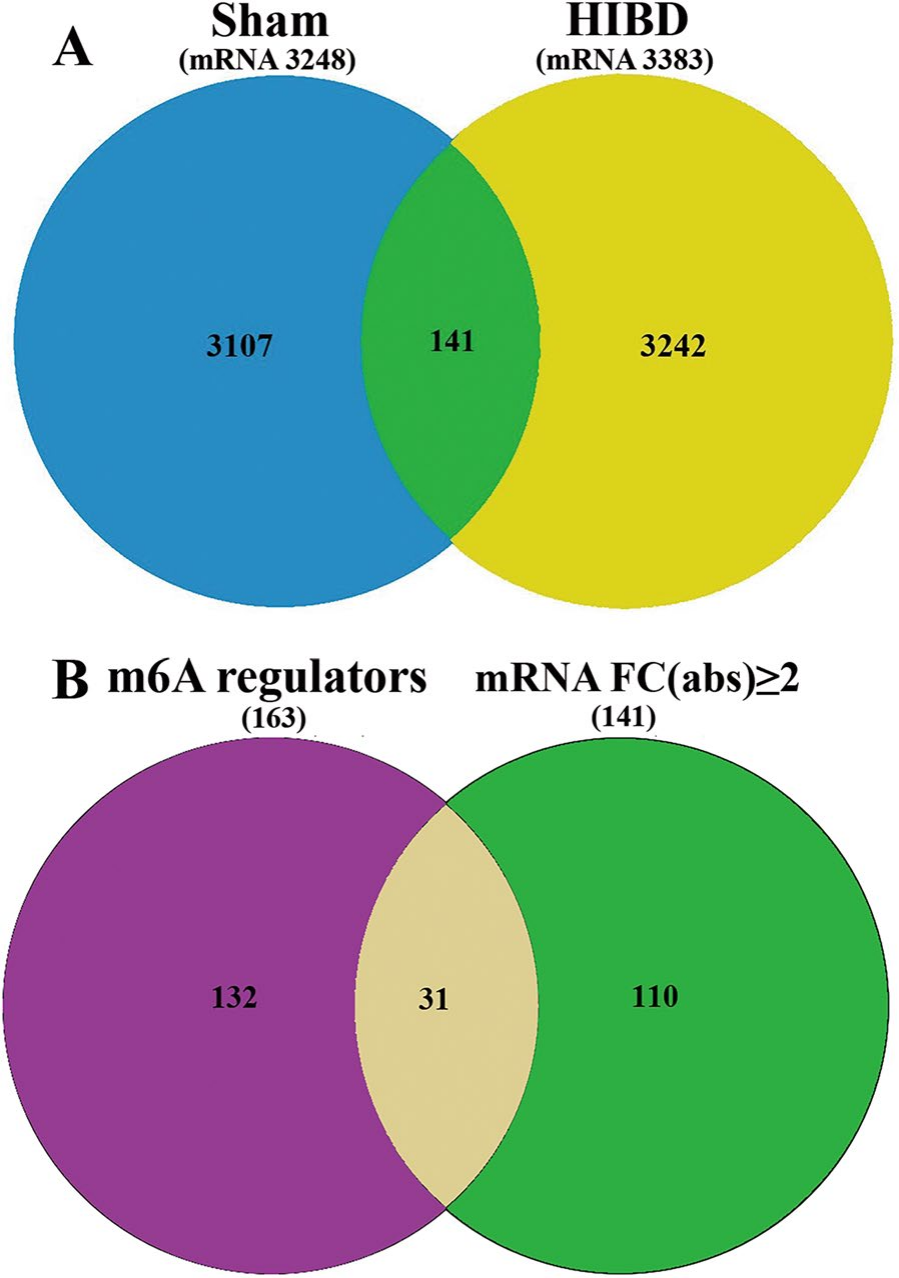
Venn diagram. **A** Screening for differentially expressed mRNA between Sham vs HIBD.** B** Screening of significantly altered m6A regulators during HIBD (FC (abs) ≥ 2, *P* < 0.05). In total, 141 differentially expressed transcripts were screened in Sham versus HIBD, and 31 significantly altered mRNA m6A regulators were identified after HIBD (FC (abs) ≥ 2, *P* < 0.05). FC (abs), absolute fold-change. Blue: total mRNA in the Sham group. Yellow: total mRNA in the HIBD group. Purple: total RNA m6A regulators. Green: mRNA with FC (abs) ≥ 2, *P* < 0.05

​At P10, cortical and hippocampus brain tissue was collected and the total RNA was then extracted for RNA sequencing. After RNA-seq analysis, we found a total of 141 mRNAs that were differentially expressed (FC (abs) ≥ 2, *P* < 0.05) in HIBD compared to the Sham group (Fig. 3A). Next, we screened the m6A regulators for the mRNAs (FC (abs) ≥ 2, *P* < 0.05) by using the RMBase v2.0 database. We found that 31 m6A regulators were significantly altered, with FC (abs) ≥ 2 and *P* < 0.05 (Fig. 3B). Collectively, these findings suggest that the mRNA m6A modification maybe involved in microglial activation for HIBD pathogenesis.

### Mettl3, Mettl14, Fto, Alkbh5, Ythdf1, and Ythdf2 are significantly downregulated in the HIBD model of microglia activation

**Fig. 4:**
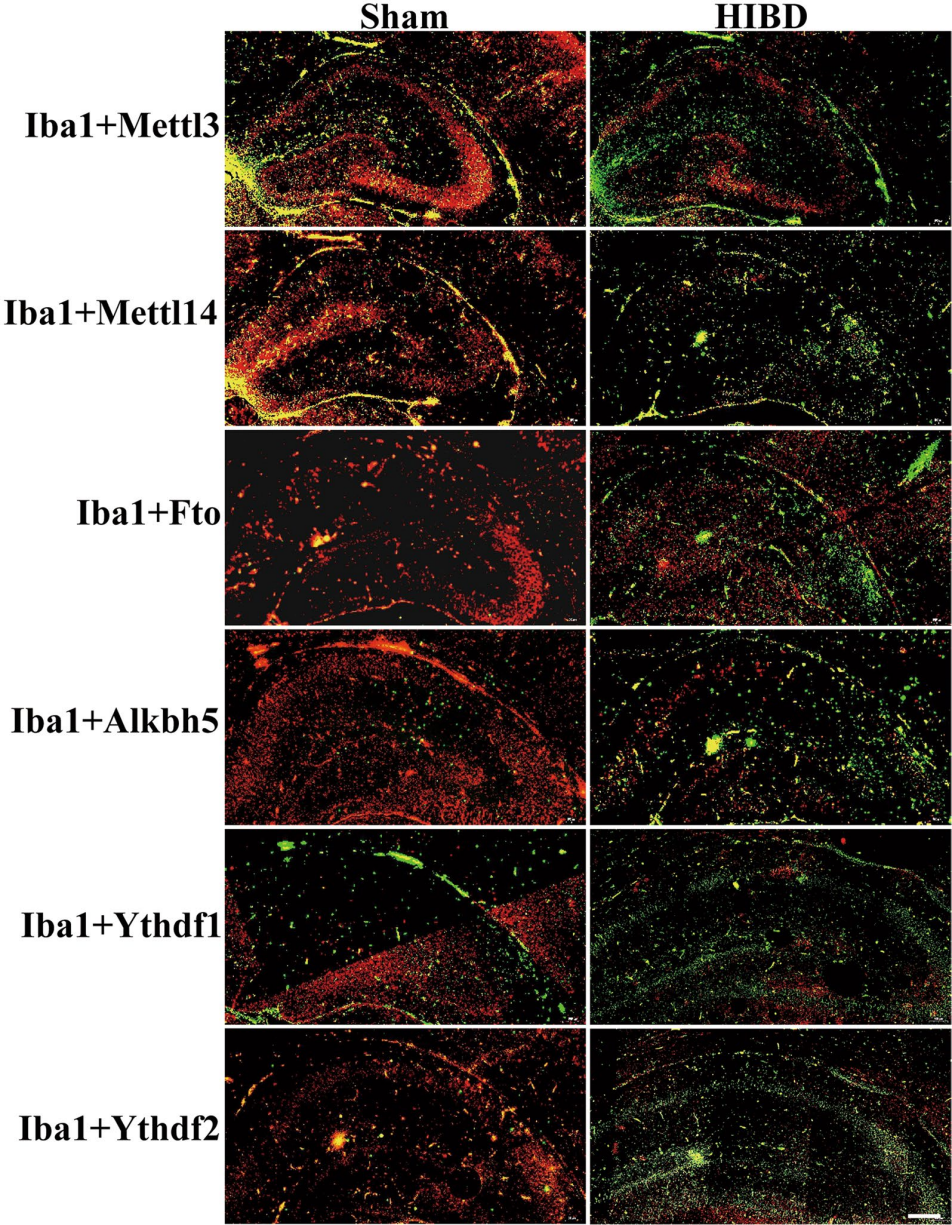
Immunofluorescence staining to validate the expression of the major m6A regulators in HIBD. The expression of Mettl3, Mettl14, Fto, Alkbh5, Ythdf1, and Ythdf2 (red fluorescence) in activated microglia (Iba1, green fluorescence) in the HIBD mouse model was detected via double immunofluorescence staining. Scale bar: 100 µm. *P < 0.05, **P < 0.01

We then selected the major m6A regulators from the 31 significantly altered mRNAs, specifically Mettl3, Mettl14, Fto, Alkbh5, Ythdf1, and Ythdf2 (Table 1) for further validation, with Mettl3 and Ythdf1 downregulated and Mettl14, Fto, Alkbh5, and Ythdf2 upregulated in sequencing results. We detected their expression in HIBD mouse tissue using double immunofluorescence staining with Iba1. The results showed that Iba1 (green, a marker for activated microglia) was significantly upregulated after HIBD when compared with the Sham group, indicating that microglia were activated in HIBD (Fig. 6). However, inconsistent with the sequencing results, we found that the expression of Mettl3, Mettl14, Fto, Alkbh5, Ythdf1, and Ythdf2 (red) in HIBD was all significantly downregulated (Fig. 4). Collectively, these findings suggest that the downregulation of these m6A regulators may play a crucial role in the regulation of microglial activation during HIBD.

**Table 1:** Sequencing and bioinformatics analysis results for the expression of the major m6A regulators after HIBD (FC (abs) ≥ 2, *P* < 0.05)

m6A regulators	*P*-value	FC (abs)*	Up/Down
Mettl3	0.0086	20	Down
Mettl14	0.0089	4.76	Up
Fto	0.0004	4.78	Up
Alkbh5	0.0009	12.9	Up
Ythdf1	0.0008	4.09	Down
Ythdf2	0.0006	2.13	Up

^*^The absolute fold-change

### Mettl3, Mettl14, Fto, Alkbh5, Ythdf1, and Ythdf2 are significantly downregulated in OGD induced microglia activation

**Fig. 5:**
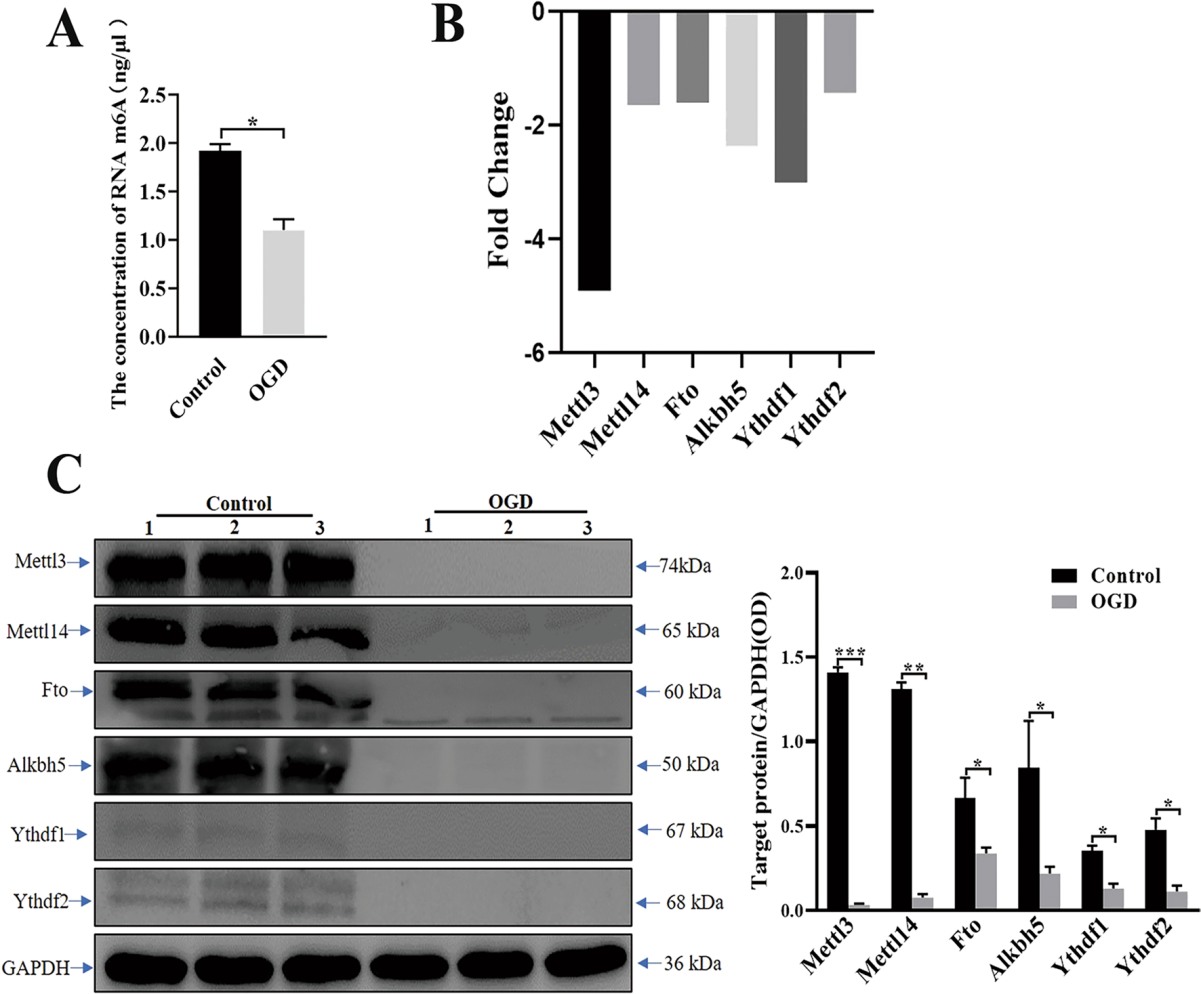
The major m6A regulators was significantly downregulated during microglial activation induced by OGD. **A** The expression of m6A level was detected in the OGD and Control group. *P = 0.0125. **B** The expression of Mettl3, Mettl14, Fto, Alkbh5, Ythdf1, and Ythdf2 was detected in microglia after OGD treatment by RT-PCR. **C** The expression of Mettl3, Mettl14, Fto, Alkbh5, Ythdf1, and Ythdf2 after OGD was detected in microglia after OGD treatment via western blot. *P < 0.05, **P < 0.01, ***P < 0.001

​Immunofluorescence staining was not able to fully quantify m6A and mRNA m6A regulatory expression. Therefore, in order to further quantify the expression of these mRNA m6A regulators in response to microglia activation after HI insult, and their relation to the whole m6A level, we cultured primary microglia followed by OGD treatment for 2 h to mimic the HI insult in vitro. Subsequently, we detected the entire m6A level by RNA m6A quantification kit and the expression of these m6A regulators by RT-PCR and Western blot. RNA m6A modification results showed the m6A level was significantly downregulated in the OGD group compared to the control group (Fig. 5A). Next, RT-PCR and western blot results showed that Mettl3, Mettl14, Fto, Alkbh5, Ythdf1, and Ythdf2 were significantly downregulated at both the mRNA (Table 2, Fig. 5B) and protein levels (Fig. 5C). Collectively, these findings suggest that the m6A levels may be associated with the downregulation of these m6A regulators, which play a crucial role in regulating microglial activation after HI insult in vivo and in vitro.

**Table 2:** RT-PCR to detect the expression of major m6A regulators

m6A regulators	RT-PCR		
	FC (abs)	Up/Down	*P*-value
Mettl3	4.91	Down	0.02
Mettl14	1.64	Down	0.04
Fto	1.59	Down	0.04
Alkbh5	2.36	Down	0.03
Ythdf1	3.01	Down	0.01
Ythdf2	1.43	Down	0.03

### GO and KEGG pathway analysis

**Fig. 6:**
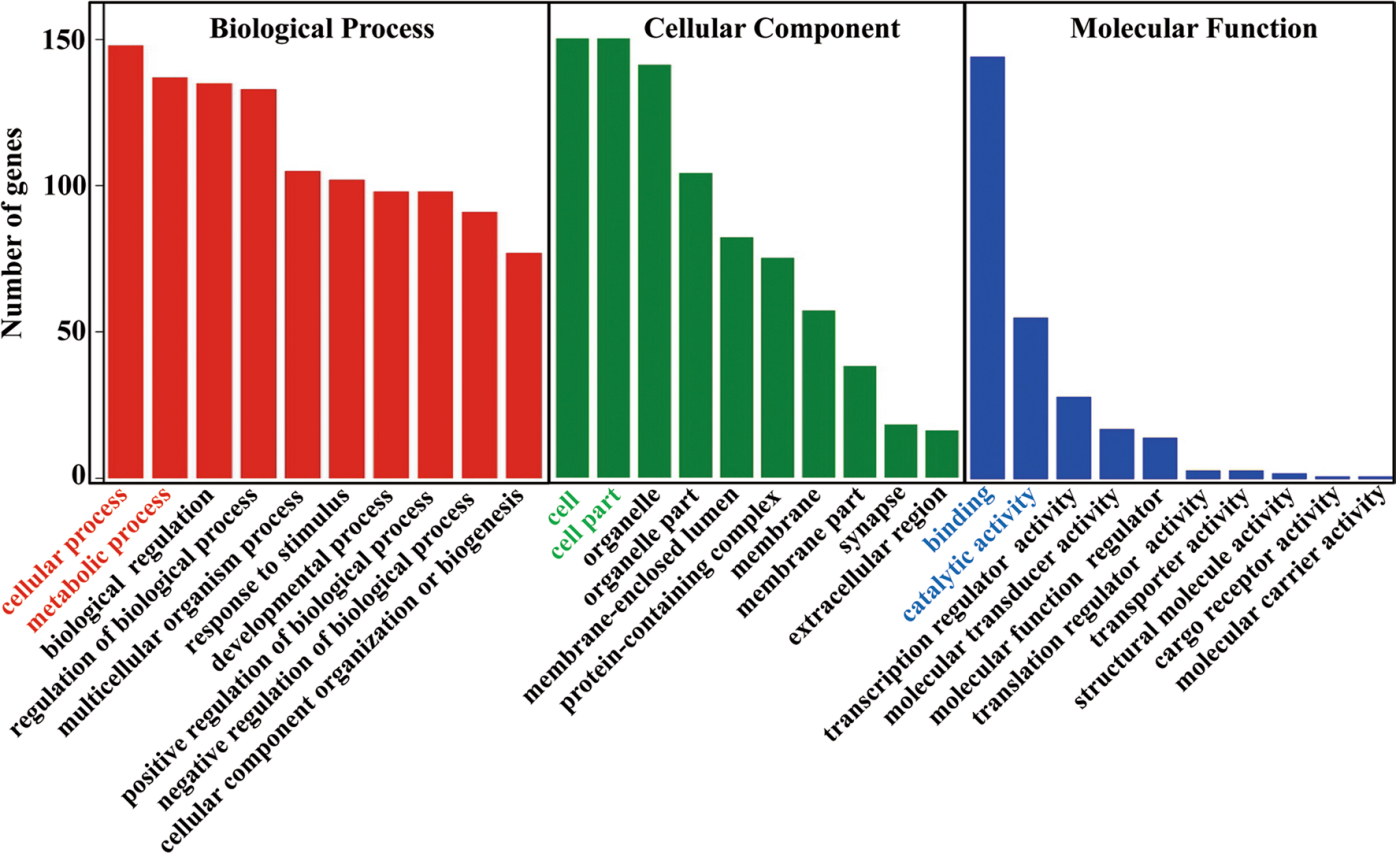
GO analysis to show the biological process, cellular component, and molecular function of these major m6A regulators. GO results showed that these major m6A regulators mainly are the component of cell and cell part, are involved in the biological processes of cellular or metabolism, and function by regulating the signal transduction pathway. GO, Gene Ontology

**Fig. 7:**
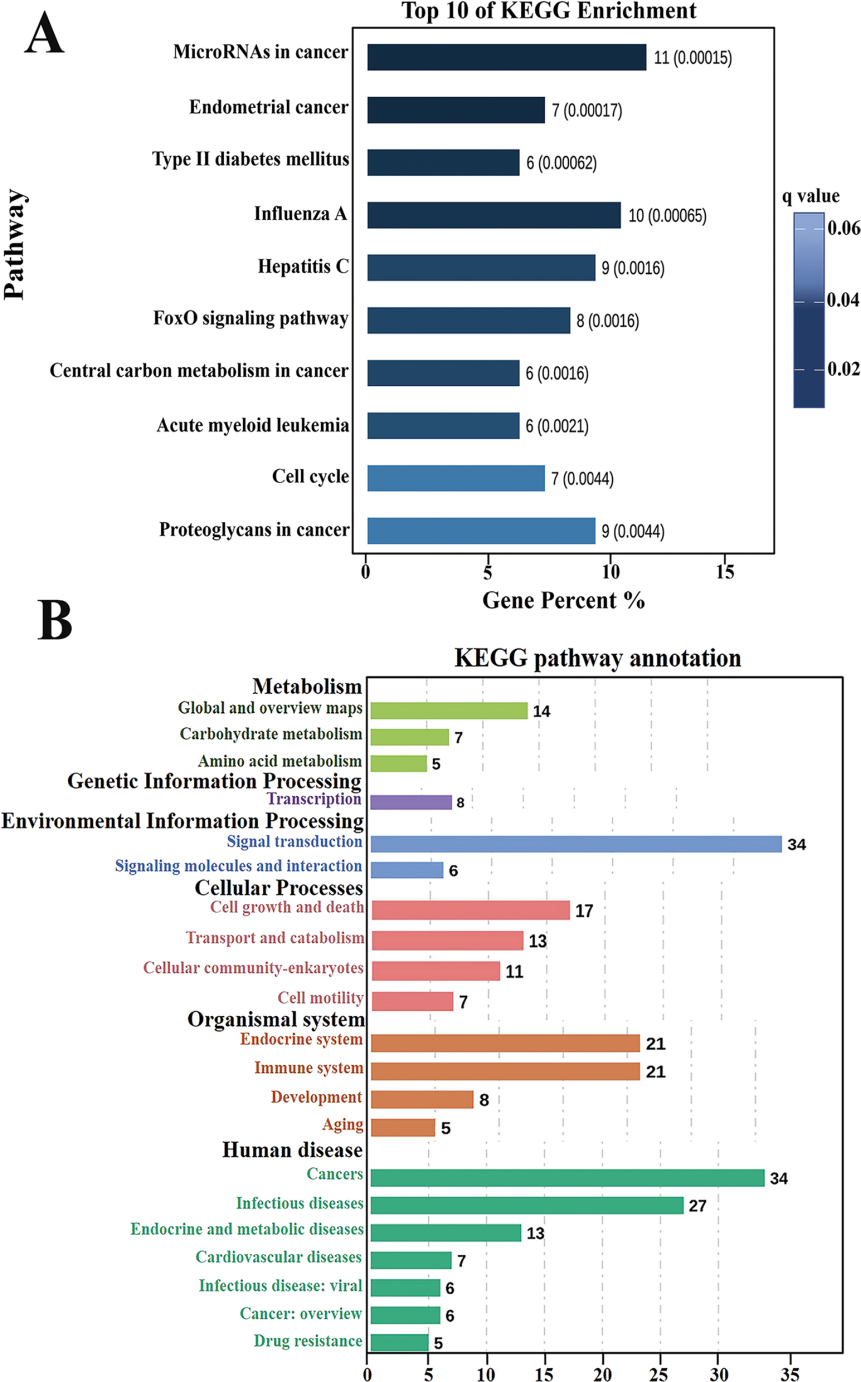
KEGG analysis to show the possible signaling pathway of these major m6A regulators. **A** Top ten KEGG pathway enrichment for the targets of these m6A regulators, which showed that their targets might be mainly micro RNAs in cancer **B** Annotation of the KEGG pathway, which showed that their targets mainly enriched in signal transduction pathway, indicating these m6A regulators might be functioned by regulating the signal transduction pathway. KEGG, Kyoto Encyclopedia of Genes and Genomes

Next, a systematic analysis of possible genetic targets for these m6A regulators was performed using the psRNATarget software, followed by GO enrichment analysis to analyze their functionality and KEGG was used to analyze the associated related pathways. GO results indicate that the target genes of these mRNA m6A regulators are primarily involved in the cellular and metabolic biological processes. Besides, we found that they primarily acted as the component of the cell and cell part, which functioned by exerting the ability of binding and catalytic activity (Fig. 6). Further, KEGG analysis indicated that they were mainly involved in the regulation of the signal transduction pathway (Fig. 7A, B).

## Discussion

In the present study, sequencing and bioinformatics analysis results found that 31 mRNA m6A regulators were differentially expressed (FC (abs) ≥ 2) in microglia in neonatal mice with HIBD, including the major regulators Mettl3, Mettl14, Fto, Alkbh5, Ythdf1, and Ythdf2, for which Mettl3 and Ythdf1 were downregulated, and the other four major regulators were upregulated. In addition, we want to elucidate the relationship between these six m6A regulators and microglia activation after HI insult, whereas in vivo HIBD models are not able to fully and accurately quantify the expression of m6A and mRNA m6A regulators specifically in microglia. Therefore, in order to further quantify the expression of these mRNA m6A regulators in response to microglia activation after HI insult, and their relation to the overall m6A level, we decided to add data from in vitro microglia models that could mimic the HI insult. First, we detached cortical and hippocampal tissue from P9 mice and isolated the primary microglia. We then treated the primary microglia with OGD for 2 h to construct an OGD model of the microglia that can mimic HI insult in vivo. Subsequently, we quantified the m6A levels using an RNA m6A quantification kit and quantified the expression of these major m6A regulators at both RNA and protein levels. However, in vitro and in vivo models, we find that all of the regulators are significantly downregulated. This inconsistent result between sequencing and validation may be due to the fact that the tissue used for sequencing may be associated with other cells than microglia, whereas the detection for validation in vivo and in vitro is conditionally localized to microglia. In general, we need to validate the sequencing data to confirm their accurate expression in vivo and in vitro. Therefore, we hypothesize that these m6A regulators are downregulated in microglia activation in HIBD and OGD. In addition, we performed a systematic analysis of possible targets for these six m6A regulators by using the psRNATarget software and then subjected them to GO and KEGG enrichment analyses. GO results highlight the involvement of Mettl3, Mettl14, Fto, Alkbh5, Ythdf1, and Ythdf2 in the cellular and metabolic processes through binding and catalytic capabilities. The KEGG pathway indicates that they function primarily in regulating the signal transduction pathways of metabolic processes. Collectively, our findings suggest a new mechanism of m6A modification in the regulation of microglial activation during HIBD, which may be mediated primarily via Mettl3, Mettl14, Fto, Alkbh5, Ythdf1, and Ythdf2. However, as we have not validated the specific function and pathways of these regulators to modulate microglial activation after HI in vitro and in vivo, future work should focus on elucidating the specific mechanisms and signaling pathways of these m6A regulators.

Several pathways have been reported to be involved in the regulation of microglial activation. For example, Wang et al. Reported that vanillin treatment could attenuate the expression of the proinflammatory cytokines IL-1β in LPS-stimulated microglia by inhibiting TLR4/NF-κB signaling, which suggested that the TLR4/NF-κB signaling pathway is involved in microglial activation [22]. In addition, one study on seizure-induced brain injury in rats revealed that everolimus exerts protective effects by reducing neuronal apoptosis and microglial activation through inhibition of the PI3K/Akt/mTOR and NF-κB/IL-6 signaling pathways, implicating both pathways in microglial activation [23]. Microglia are rapidly activated during intracerebral hemorrhage and have the capacity to resist hemin toxicity [23]. One study reported that microglia activation via LPS pre-treatment markedly reduced their vulnerability to hemin toxicity in vitro and in vivo [24]. This is because LPS upregulates the expression of inducible NO synthase and heme oxygenase, the rate-limiting enzymes of heme degradation, in microglia [24]. Mechanistically, this effect was mediated via inhibition of the JNK and p38 MAPK pathways, which in turn suggested their involvement in microglial activation [24]. Furthermore, adenosine 5'-monophosphate-activated protein kinase (AMPK) is associated with cellular inflammation [25, 26]. Treatment of LPS-activated BV2 inflammation with ENERGI-F704 decreased the production of IL-6, TNF-α, and NO by regulating AMPK signaling [25]. In addition, Notch, STAT, CREB, PPAR-γ, and other pathways have also been reported to regulate microglial activation either directly or indirectly [7].

The m6A modification of RNA has also been described to modulate microglial activation via the aforementioned listed pathways. In LPS-induced microglial inflammation, Mettl3 expression was upregulated, in parallel to inflammatory cytokines (IL-1β, IL-6, IL-18, and TNF-α) and inflammatory proteins (TRAF6 and NF-κB) [27]. Further, Mettl3 levels were positively correlated with those of TRAF6, and the two proteins could directly interact [27]. Overexpression of Mettl3 promoted activation of the TRAF6-NF-κB pathway in an m6A-dependent manner, and inhibition of NF-κB attenuated Mettl3-mediated microglial activation [27]. This study indicated that the m6A methyltransferase Mettl3 promotes LPS-induced microglial activation through TRAF6/NF-κB signaling [27]. In contrast, we find that Mettl3 is significantly down-regulated in hypoxia-induced microglial activation. These different results may be due to the different models used for Mettl3 detection, as well as different targets and mechanisms for the function of Mettl3. Another work in LPS-activated microglia revealed distinct m6A methylation patterns and identified insulin-like growth factor 2 mRNA binding protein 1 (Igf2bp1) as the most significantly up-regulated m6A factor as well as ceruloplasmin (Cp) and guanylate-binding protein 11 (Gbp11) as its key target mRNAs [28]. Gbp11 and Cp are proinflammatory proteins that could significantly upregulate the expression of TNFα, IL-1β, and CD68 proteins in LPS-stimulated microglia [28]. Cp is an enzyme with critical functions in iron homeostasis and inflammation [29]. It has been reported that Cp can stimulate MAPK and NF-κB signaling and, in turn, activate BV2 microglial cells [30, 31]. In addition, conditional deficiency of m6A methyltransferase Mettl14 in the substantia nigra reduced tyrosine hydroxylase expression and enhanced microglia activation, which was strongly related to m6A mRNAs nuclear receptor-related protein 1 (Nurr1), pituitary homeobox 3, and en-grailed1 [32]. Nurr1 belongs to the orphan nuclear receptor 4 superfamily expressed by microglia [33]. Activation of Nurr1 inhibits the phagocytic function of BV-2 cells via the ERK1/2 signaling pathway [34].

## Conclusion

Taken together, both of the previous existing knowledge and our current study suggest that mRNA m6A may regulate microglial activation via the aforementioned pathways or molecular effectors following HI. While our findings from GO and KEGG analyses highlight the involvement of mRNA m6A modifications in the regulation of microglial activation during HIBD, mediated by Mettl3, Mettl14, Fto, Alkbh5, Ythdf1 and Ythdf2, through their influence on cellular metabolic processes by exerting binding or catalytic activity. However, the exact mechanism remains to be determined in future studies. Furthermore, as we found significant expression alterations in these major m6A regulators during microglia activation, we hypothesize their promising potential as diagnostic or prognostic biomarkers, as well as therapeutic targets for microglia activation-induced disorders.

## Materials and methods

### Animals and models

All animal experiments were approved by the Sichuan University Committee on Animal Research and complied with the ARRIVE guidelines (approval no. WCSUH21-2018-034). Post-natal day 9 (P9) C57BL/6 mice (average weight, 5.3 ± 0.63 g) were purchased from Sichuan Dashuo Animal Science and Technology Co., Ltd. (Chengdu, China).

All the mice were fed with breast milk and maintained under a 12 h light/12 h dark cycle. The neonatal mice model of HIBD was established as previously described (Rice et al., 1981). The process of HIBD modeling is as follows: All of the neonatal mice at P9 (male and female) were randomly divided into HIBD and Sham groups (n = 6). And then anesthetized with isoflurane (induction concentration: 4%, maintenance concentration: 2%). For mice in the HIBD group, an incision of approximately 1 cm was made in the neck skin. The right carotid artery was exposed and ligated with a 6–0 suture after separation from the glands and muscle tissue. After surgery, mice were returned to the incubator at a constant temperature (37.0 °C) for 30 min for recovery. Subsequently, the mice were placed in a hypoxia cabin with the condition of 8% O_2_ and 92% N_2_ at 37 °C and with a gas flow rate of 3 L/min for 1.5 h in order to induce HIBD. For the Sham group, mice were only subjected to neck incision for dissociation of the right carotid artery, without ligation or hypoxia. Finally, all mice were returned to their cages and sacrificed at P10.

### Hematoxylin & eosin (H&E) staining

At P10, Mice were killed and the whole brain was removed to 4% paraformaldehyde for a fix. After two days of fixing, the brain tissue was embedded in paraffin and sectioned into 4-μm-thick slices. Subsequently, the slices were deparaffinized in xylene and rehydrated through graded alcohol solutions (100%, 5 min × 2; 95%, 5 min × 1; 85%, 2 min × 1; 70%, 2 min × 1). Next, the slices were washed with distilled water two times and each time for 5 min. Finally, the slices were performed with hematoxylin staining for 5 min, discarded the hematoxylin and rinsed with running water for 5 s, differentiated in 1% hydrochloric alcohol for 20–30 s and then rinsed with running water for 5 s, gradient alcohol dehydration and sealed with a cover glass. The HE results were observed under a light microscope (Olympus, Tokyo, Japan) to analyze the pathological characteristics (morphology, inflammatory cell infiltration, cell edema, and death) of the cortex and hippocampus. Six animals were analyzed in each group.

### RNA-seq

At P10, the mice in each group (n = 6) were anesthetized with isoflurane, and their brains were removed. We then detached the cortex and hippocampus, from which we then extracted total RNA (#AM1561 from Ambion^®^) for RNA-seq (Beijing Novo Gene Technology Co., LTD). The procedure for RNA sequencing was as follows: Total RNA was quantified using the Nanodrop ND-2000 (Thermo Scientific), and RNA integrity was assessed using Agilent Bioanalyzer 2100 (Agilent Technologies). The criterion for the quality of RNA can be assessed by the RNA integrity number (RIN) and the ratio of 28S/18S. RIN ≥ 7 and 28S/18S ≥ 0.7 indicate that the RNA is feasible for RNA-Array. Total RNA was dephosphorylated, denatured, and then labeled with Cyanine-3-CTP. After purification, the labeled RNAs were hybridized onto the Agilent mice RNA microarray. In-depth data analysis was provided by Beijing Novo Gene Technology Co., LTD.

### Screening differentially expressed m6A regulators

First, for the RNA data analysis, feature extraction software (version 10.7.1.1, Agilent Technologies) and gene spring (version 13.1, Agilent Technologies) were employed to complete the basic analysis, and the raw data were normalized with the quantile algorithm. Differentially expressed mRNAs were identified via the absolute fold-change (FC (abs)) and t-test, where FC (abs) ≥ 1.5 and *P* < 0.05 indicated a significant change. Next, we screened for all m6A regulators in RMBase v2.0 (http://rna.sysu.edu.cn/rmbase/), a database for studying m6A RNA modifications. Among the differentially expressed transcripts, we obtained 31 significantly altered mRNA m6A regulators, with FC (abs) ≥ 2.0 and *P* < 0.05. Among these, Mettl3, Mettl14, Fto, Alkbh5, Ythdf1, and Ythdf2 have been reported as major regulators of the m6A modification [13]. Thus, we subjected these to further analysis.

### OGD model of primary microglia

Primary microglia from the brains of P9 mice (n = 6) were cultured as follows: (1) Culture plate coating: add 10 μg/ml polylysine to six-well plates, incubate at 37 ℃ for 4 h, suction out excess lysine, wash 3 times with sterilized pure water, and dry in a biosafety cabinet for later use. (2) Brain extraction: P9 mice, male or female, were sacrificed after hypothermia anesthesia, soaked in a 75% alcohol beaker for disinfection, and decapitated. The scalp and skull were cut in layers under sterile conditions, and the whole brain was removed and placed in precooled PBS Petri dishes. (3) Isolation: The cerebral cortex and hippocampus were isolated and the vascular membrane was removed. The obtained tissue was placed into a precooled DMEM high glucose (4 ml) culture dish, cut into 1 mm^3^ pieces by ophthalmic scissors, and prepared for digestion. (4) Digestion: 1 ml papain (working concentration 2 mg/ml) was added, 50 ul DNase (1 U/ul in aliquots, 30 U/ml) was covered and digested in a 5% CO_2_ incubator at 37 °C for 30 min, during which time the plates were shaken gently 3 times. (5) Filtration: tilt the culture dish, keep the culture dish for 2–3 min, precipitate the tissue block, suck out the upper digester, retain the digested tissue block, add the growth medium (containing FBS) to terminate the digestion, gently blew it and then stood. After the bulk tissue was precipitated, the supernatant was sucked and filtered with a 200-mesh screen (70um), and the cell filtrate was collected. (6) Inoculation: growth medium (50 ml: 45 ml DMEM high glucose + 5 ml FBS + 500 ul double antibody) suspend cells, gently blow to the single cell suspension. According to the counting results, 1.5 to 2 × 10^7^ cells were adjusted to be seeded into T75 culture flasks. (7). Liquid exchange: 2–3 days after inoculation, the culture flask was placed on a shaker when the mixed cells were fully grown. The temperature was set at 37 °C and the rotation speed was 180 rpm for 30–40 min. Subsequently, the culture medium was collected into a centrifuge tube and centrifuged at 1000 rpm for 10–15 min, and then the precipitated cells were microglia.

To this end, primary microglia were removed from the medium (DMEM + FBS + penicillin–streptomycin), washed three times with PBS, and added to the pretreated sugar-free and serum-free DMEM medium to simulate the cell ischemia state. And then maintained at 37 °C in hypoxic cabins with conditions (94% N_2_, 5% CO_2_, and 1% O_2_) for 2 h to establish the OGD group in vitro. The corresponding control group was cultured with DMEM medium (DMEM + FBS + penicillin–streptomycin) at the conditions of 37 ℃, 95% O_2_, and 5% CO_2_. After OGD, microglia were collected, and then extracted the total RNA and protein by RNA extraction kit (#AM1561 from Ambion^®^) and protein extraction kit (cat. no. BB-31227-1; Chengdu bei Bo; http://beibokit.com/), respectively.

### Detection of m6A RNA expression in cultured microglia

The EpiQuik m6A RNA methylation quantitative detection kit (ab185912, colorimetric, article no. P-9005-48) was used to detect the expression of RNA m6A after OGD. Experimental procedures were performed according to the manufacturer’s instructions.

### RT-PCR

Total RNA was extracted from microglia (#AM1561 from Ambion^®^) and then subjected to reverse transcription, followed by PCR. mRNA expression was calculated using the 2-ΔΔCt method and normalized to that of GAPDH. Primer sequence information for Mettl3, Mettl14, Fto, Alkbh5, Ythdf1, and Ythdf2 is provided in Additional file 3. Supplementary Tables: Table S1.

### Immunofluorescence staining

To validate the expression of major regulators of Mettl3, Mettl14, Fto, Alkbh5, Ythdf1, and Ythdf2 in microglia in vivo, we performed double immunofluorescence staining with Iba1. The brain tissues of mice were frozen, sectioned, blocked with goat serum for 1 h, and incubated with primary antibody at 4 °C overnight. The next day, sections were washed with PBS, where a fluorescently-labeled secondary antibody was added and incubated in a wet box at room temperature for 2 h. After washing with PBS, DAPI was added to sections and incubated in the dark for 5 min. Finally, the sections were again washed with PBS, and an anti-fluorescence quenching agent was added. Fluorescence images were obtained using a confocal laser-scanning microscope (Olympus, Japan) and FV-ASW-3.1 software (Olympus). Antibody information is provided in Additional file 3. Supplementary Tables: Table S2.

### Western blot

Total protein was extracted from microglia (cat. no. BB-31227-1; Chengdu bei Bo; http://beibokit.com/), and protein concentration was determined using the BCA protein assay kit (Pierce) with bovine serum albumin as the standard. SDS–polyacrylamide gel (10%) was used to separate target proteins (Mettl3, Mettl14, Fto, Alkbh5, Ythdf1, and Ythdf2) and the internal reference protein GAPDH. Finally, immunoreactive signal bands were quantified using ImageJ software. The optical densities (OD) ratio of the target protein to the internal reference protein was used to generate a statistical graph. All experiments were repeated at least three times to ensure the reproducibility of the results. Antibody information is provided in Additional file 3. Supplementary Tables: S2.

### GO and KEGG enrichment analyses

Further systematic analysis of possible gene targets for the differentially expressed m6A regulators was conducted using psRNATarget software and then subjected to GO enrichment analysis to analyze their functions (http://www.geneontology.org), which includes the biological process, cellular component, and molecular function categories. KEGG was used to analyze related pathways. GO and KEGG enrichment analyses were carried out using the Metascape database (https://metascape.org/gp/index.html#/main/step1). *P* < 0.05 indicated significantly enriched terms.

### Statistical analysis

Statistical analysis was performed using SPSS 23.0. Data of relative OD of immunoblots are presented as the mean ± standard deviation (SD). After determining the normality and variance homogeneity of the data, we performed between-group comparisons using Student's t-test. Statistical significance was assigned as *P < 0.05, **P < 0.01, ***P < 0.001.

### Supplementary Information

**Additional file 1**. **Figure S1**. Illustration of microglia activation in HIBD.

**Additional file 2. Figure S2**. Schematic diagram of mRNA m6A modification mechanism.

**Additional file 3:** Supplementary Tables for the information of the primer sequence and antibodies in this study.

## Data Availability

The dataset(s) supporting the conclusions of this article is(are) included within the article (and its additional file(s)).

## Abbreviations

HIBD: Hypoxic-ischemic brain damage
OGD: Oxygen and glucose deprivation
HI: Hypoxia–ischemia
TNF-α: Tumor necrosis factor-α
IL-1β: Interleukin-1β
IL-6: Interleukin-6
IFN-γ: Interferon-γ
Arg-1: Arginase-1
m6A: M6-methyladenosine
Mettl3/Mettl14: Methyltransferase 13/14
–CH_3_: Methyl groups
GO: Gene Ontology
KEGG: Kyoto Encyclopedia of Genes and Genomes
P9: Post-natal day 9
FC (abs): The absolute fold-change
OD: Optical densities
SD: Mean ± standard deviation
AMPK: Adenosine 5′-monophosphate-activated protein kinase
Igf2bp1: Like growth factor 2 mRNA binding protein 1
Cp: Ceruloplasmin
Gbp11: Guanylate-binding protein 11
Nurr1: Nuclear receptor-related protein 1
