# Supplementary figures and images for: The RNA m6A modification might participate in microglial activation during hypoxic–ischemic brain damage in neonatal mice
Source: Hum Genomics. 2023 Aug 25;17:78. doi: 10.1186/s40246-023-00527-y (PMC10463984; doi:10.1186/s40246-023-00527-y)

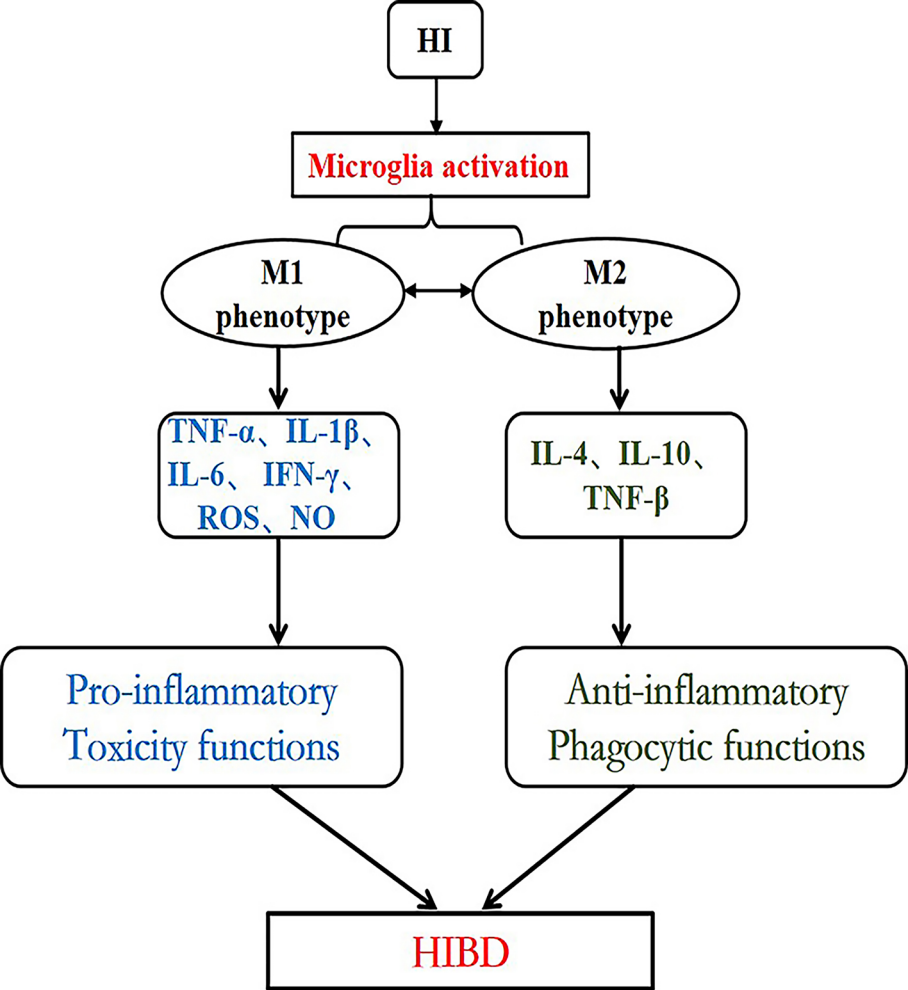

Supplement: Supplementary file 1 — Additional file 1. Figure S1. Illustration of microglia activation in HIBD. [file 40246_2023_527_MOESM1_ESM.pdf]

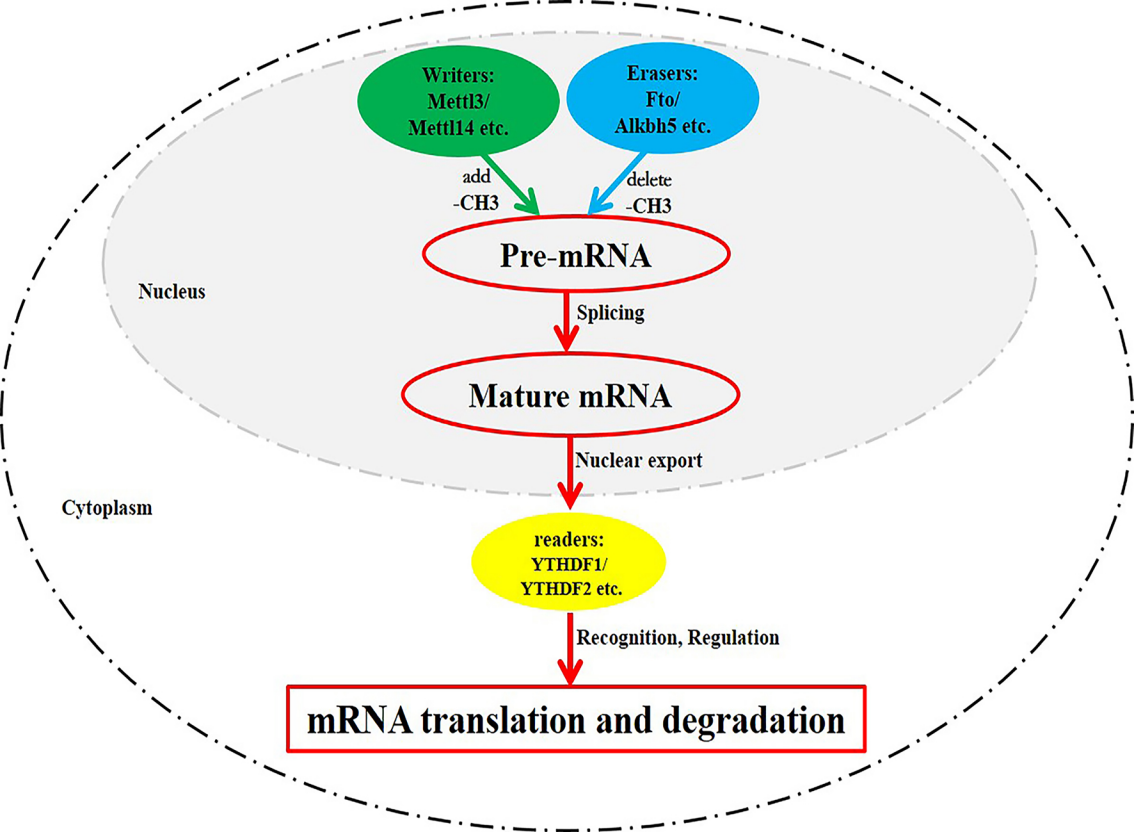

Supplement: Supplementary file 2 — Additional file 2. Figure S2. Schematic diagram of mRNA m6A modification mechanism. [file 40246_2023_527_MOESM2_ESM.pdf]
