# Supplementary material for: The RNA m6A modification might participate in microglial activation during hypoxic–ischemic brain damage in neonatal mice
Source: Hum Genomics. 2023 Aug 25;17:78. doi: 10.1186/s40246-023-00527-y (PMC10463984; doi:10.1186/s40246-023-00527-y)
Supplement: Supplementary file 3 — Additional file 3: Supplementary Tables for the information of the primer sequence and antibodies in this study. [file 40246_2023_527_MOESM3_ESM.docx]

**Supplementary Tables**

**Supplementary Table 1.** Primer sequence information for Mettl3, Mettl14, Fto, Alkbh5, Ythdf1, Ythdf2, and GAPDH

| **mRNA name** | **Sense primer** | **Anti-sense primer** |
| --- | --- | --- |
| Mettl3 | 5'CTCTGGGCACTTGGATTTA 3' | 5'CACGGGACTATCACTACGG 3' |
| Mettl14 | 5'TCAAAGGAACCGTGAAGCG3' | 5'CCAGCCTGGCCTGATAGTG 3' |
| Fto | 5'GAGCAGAGCAGCCTACAAC3' | 5'TGGACTCGTCCTCACTTTC 3' |
| Alkbh5 | 5'GGTGTCGGAACCTGTGCTTT3' | 5'TGGAGCTGCTCAGGGATTTT3' |
| Ythdf1 | 5'GGACATTGGTACTTGGGATA 3' | 5'CTCTGATACTGTGGCTGGAC3' |
| Ythdf2 | 5'CCCTCACAGGCTTTGGTTCA3' | 5'GGCTGCGTCTGTTGCCCTAC3' |
| GAPDH | 5'GGTCCCAGCTTAGGTTCAT 3' | 5'CAATCTCCACTTTGCCACT 3' |

**Supplementary Table 2.** Information on the antibodies used in immunofluorescence and western blot experiments

|  | **Antibody name** | | **Host species** | **Dilution** | **Catalog #** |
| --- | --- | --- | --- | --- | --- |
| **Western blot** | Primary antibody | Mettl3 | Rabbit | 1:200 | NBP3-03290 |
|  |  | Mettl14 | Rabbit | 1:250 | NBP1-81392 |
|  |  | Fto | Rabbit | 1:100 | NBP1-77021 |
|  |  | Alkbh5 | Rabbit | 1:200 | NBP1-82188 |
|  |  | Ythdf1 | Rabbit | 1:3000 | 17479-1-AP |
|  |  | Ythdf2 | Rabbit | 1:5000 | 24744-1-AP |
|  |  | GAPDH | Mouse | 1: 5000 | Santa Cruz Biotechnology |
|  | Secondary antibody | Peroxidase conjugated | Goat anti-rabbit /mouse IgG | 1: 5000 | Santa Cruz Biotechnology |
| **Immunofluorescence**  **Immunofluorescence** | Antibody name | | Host species | Dilution | Catalog # |
|  | Primary antibody | Mettl3 | Rabbit | 1:100 | NBP3-03290 |
|  |  | Mettl14 | Rabbit | 1:500 | NBP1-81392 |
|  |  | Fto | Rabbit | 1:100 | NBP1-77021 |
|  |  | Alkbh5 | Rabbit | 1:200 | NBP1-82188 |
|  |  | Ythdf1 | Rabbit | 1:500 | 17479-1-AP |
|  |  | Ythdf2 | Rabbit | 1:500 | 24744-1-AP |
|  |  | Iba1 | Goat | 1:500 | MAB345 |
|  | Secondary antibody | Cy3/48 conjugated | Donkey/goat anti-rabbit/ mouse IgG | 1:500 | Jackson |
